# AI in orthodontic treatment planning: a systematic review comparing learning approaches

**DOI:** 10.1186/s12903-026-08854-x

**Published:** 2026-06-12

**Authors:** Martin Baxmann, Márton Zsoldos, Krisztina Kárpáti

**Affiliations:** Department of Orthodontics and Paediatric Dentistry, Faculty of Dentistry, University of Szeged, Szeged, H-6720 Hungary

**Keywords:** AI in orthodontics, Case-based learning, Rule-based learning, Treatment planning, Clinical decision support, Artificial intelligence

## Abstract

**Background:**

Artificial intelligence has increasingly been applied to orthodontic diagnosis and treatment planning. Clinical decision-making in orthodontics is complex and often varies among practitioners, particularly for decisions such as tooth extraction, skeletal classification, and selection of treatment modality. This systematic review aimed to evaluate how artificial intelligence and knowledge-based systems have been developed and assessed for orthodontic treatment planning, to compare their performance with expert clinicians, and to examine how different learning approaches influence accuracy, interpretability, and potential clinical integration.

**Methods:**

A comprehensive search of PubMed, Embase, Scopus, Web of Science, Cochrane Library, and IEEE Xplore was conducted from database inception to 15 October 2025 without language or date restrictions. Eligible studies evaluated artificial intelligence–based or knowledge-based systems using real orthodontic patient data to support diagnosis or treatment planning. Two reviewers independently screened records, extracted data, and assessed risk of bias using the ROBINS-I tool. Due to heterogeneity in study designs, decision tasks, and outcome measures, findings were synthesized narratively rather than through meta-analysis.

**Results:**

Nineteen studies met inclusion criteria. Most investigated supervised machine-learning models for extraction decisions, skeletal classification, or multi-step treatment planning, while others evaluated rule-based, fuzzy, Bayesian, or hybrid systems. Reported accuracies frequently exceeded 80%, and in some studies surpassed 90% when compared with expert decisions. Knowledge-based systems, including rule-based, fuzzy, and Bayesian/probabilistic approaches, offered greater transparency in reasoning, whereas data-driven models often demonstrated higher discriminative performance. Overall risk of bias was predominantly moderate to serious, largely due to retrospective designs, single-center samples, limited external validation, and reliance on expert opinion as the reference standard.

**Conclusions:**

Artificial intelligence systems can approximate expert orthodontic decision-making for defined diagnostic and planning tasks and show promise as clinical decision-support tools. However, current evidence is limited by methodological weaknesses and lack of prospective validation. These systems should presently be considered adjunctive aids rather than autonomous planners. Robust multicenter studies with external validation are needed before routine clinical implementation can be recommended.

**Trial registration:**

PROSPERO 1025581. Registered prospectively.

**Supplementary Information:**

The online version contains supplementary material available at https://doi.org/10.1186/s12903-026-08854-x.

## Background

Artificial intelligence (AI) has moved rapidly from experimental proof-of-concept to practical support tool in many areas of dentistry, and orthodontics is no exception. Clinical decision-making in orthodontics is inherently complex: treatment planning requires the integration of skeletal and dental relationships, growth potential, soft-tissue considerations, patient preferences and multiple therapeutic options, often in the context of incomplete or uncertain information [1–6]. Considerable inter- and intra-clinician variability has been reported in key decisions such as extraction versus non-extraction therapy, growth modification versus camouflage or surgery, and the management of impacted canines and other local problems [7–9]. AI-based clinical decision support systems (CDSS) have been proposed as a way to reduce this variability, standardize reasoning, and extend expert-level decision-making to a wider range of practitioners [10–12].

Early CDSS in orthodontics were predominantly knowledge-based, including rule-based expert systems, fuzzy systems, and Bayesian/probabilistic models in which clinical rules, uncertainty, probabilities, or causal assumptions were encoded explicitly by domain experts [13]. Examples include Bayesian networks used to explore how radiographic and demographic features influence the management of impacted maxillary canines, and to visualize potential causal pathways between diagnostic variables and treatment duration [14–16]. In parallel, data-driven approaches have emerged that learn directly from large collections of treated cases. Contemporary machine-learning models trained on hundreds of orthodontic records have been designed to predict skeletal classification, extraction decisions, and broad treatment strategies such as growth modulation, camouflage or orthognathic surgery, often achieving levels of agreement with expert orthodontists in the range of 80–90% [17–20]. These systems, which include artificial neural networks, ensemble tree-based models and other statistical learners, generate predictions from patterns mined from previous clinical experience.

Although narrative reviews have described the expanding role of AI in orthodontics and highlighted applications such as cephalometric landmark detection, image segmentation and workflow automation, a focused synthesis of AI-driven decision support for treatment planning is still lacking [21–25]. Existing primary studies vary widely in their clinical questions, ranging from prediction of extraction need and treatment outcome to the planning of impacted canine traction or the stratification of skeletal Class II and III cases [26–30]. They also span a spectrum of learning paradigms, from knowledge-based systems, including rule-based, fuzzy, and Bayesian approaches, to data-driven machine-learning models and hybrid frameworks that combine explicit reasoning with statistical prediction [17, 31, 32]. As a result, the evidence base is fragmented, and it is unclear how design choices in knowledge representation—rule-based versus case-based or other data-driven approaches—influence diagnostic performance, robustness, transparency and clinical usability [20, 25, 33].

A rigorous synthesis is therefore needed to map and critically appraise the current landscape of AI-based CDSS for orthodontic treatment planning, and to clarify the extent to which these tools can support or augment clinician judgement. This systematic review addresses three interrelated questions: which types of AI-driven clinical decision support systems have been developed and evaluated for orthodontic treatment planning; how accurate and reliable are these systems, compared with expert orthodontists or other reference standards, across different decision tasks; and in what ways do their underlying learning paradigms - knowledge-based systems, data-driven machine-learning models, case-based reasoning systems, and hybrid approaches - shape their performance, interpretability and potential for integration into everyday orthodontic practice.

## Methods

This study was conducted as a systematic review of primary research on artificial intelligence–based decision support for orthodontic diagnosis and treatment planning, following current reporting standards for systematic reviews. A protocol prespecifying the review question, eligibility criteria, risk-of-bias methods and synthesis plan was registered in PROSPERO (1025581) before study selection. During scoping searches it became clear that virtually no studies directly compared case-based learning with rule-based learning within the same trial. The protocol was therefore amended, before full-text screening, to include all AI-based decision-support systems for orthodontic decision-making, irrespective of whether the underlying approach was knowledge-based, including rule-based, fuzzy, or Bayesian/probabilistic systems; data-driven machine learning; case-based reasoning; or a hybrid framework. The review question was reformulated to describe and compare the performance and characteristics of AI-driven decision-support systems used for orthodontic diagnosis and treatment planning, and to explore how these systems relate to traditional clinician-driven decision-making. AI was used to proofread and improve the readability of the manuscript.

### Eligibility criteria and study selection

Eligible studies used individual orthodontic patient data to develop, validate or evaluate an AI-based system that generated a diagnostic classification or treatment-planning recommendation. Populations comprised patients receiving, or being considered for, orthodontic treatment; their clinical, radiographic or cephalometric records were used as model inputs. Interventions were knowledge-based or data-driven AI systems designed to support decisions such as extraction versus non-extraction, choice of growth modification, camouflage or orthognathic surgery, management of impacted canines, selection of headgear type, prioritization of treatment need or other case-level planning tasks. Comparators included expert orthodontists or general dentists, guideline-based indices, alternative AI models, or internal reference standards. Studies without an explicit external comparator were eligible when internal validation using standard performance metrics was reported and the system was intended for real clinical decision support rather than purely technical image analysis. These criteria reflect a Population–Intervention–Comparator–Outcome (PICO) framework, encompassing orthodontic patients, AI-based decision-support systems, relevant clinical or model-based comparators, and performance or clinical utility outcomes.

Primary outcomes were measures of diagnostic or planning performance, including overall accuracy, sensitivity, specificity, area under the receiver-operating characteristic curve, agreement statistics and misclassification rates. Secondary outcomes included any indicators of clinical utility or implementation, such as changes in clinicians’ decisions, user acceptance, or perceived usefulness in practice or education. Eligible designs comprised retrospective or prospective observational cohorts, diagnostic accuracy studies, cross-sectional evaluations of AI-generated plans and modelling studies embedded in clinical datasets. Narrative reviews, editorials, opinion pieces, conference abstracts without full text, proof-of-concept simulations using only synthetic data, and technical image-processing studies without an explicit decision-support objective were excluded. No restrictions were applied to year, language or geographic setting, provided that full text was available.

### Information sources and search strategy

Electronic searches were undertaken in PubMed (via MEDLINE), Cochrane Library, Embase, Scopus, Web of Science and IEEE Xplore from database inception to 15 October 2025. Search terms were adapted to each database and combined controlled vocabulary (for example MeSH and Emtree) with free-text keywords for three core concepts: orthodontics, artificial intelligence or clinical decision support, and diagnostic or treatment-planning tasks. No language or date limits were applied. For transparency, the complete PubMed strategy was:

( “Orthodontics“[Mesh] OR “Orthognathic Surgery“[Mesh] OR “Cephalometry“[Mesh] OR orthodontic*[tiab] OR malocclusion*[tiab] OR “orthodontic diagnosis“[tiab] OR “orthodontic treatment“[tiab] OR “orthodontic treatment planning“[tiab] ) AND ( “Artificial Intelligence“[Mesh] OR “Machine Learning“[Mesh] OR “Neural Networks, Computer“[Mesh] OR “Decision Support Systems, Clinical“[Mesh] OR “Expert Systems“[Mesh] OR “Bayes Theorem“[Mesh] OR “Fuzzy Logic“[Mesh] OR “Pattern Recognition, Automated“[Mesh] OR “Data Mining“[Mesh] OR “artificial intelligence“[tiab] OR “machine learning“[tiab] OR “deep learning“[tiab] OR “neural network*“[tiab] OR “expert system*“[tiab] OR “knowledge-based“[tiab] OR “knowledge based“[tiab] OR “Bayesian network*“[tiab] OR “fuzzy“[tiab] OR “clinical decision support“[tiab] OR “decision support“[tiab] ).

Search strategies for the other databases followed the same conceptual structure, with database-specific subject headings and syntax, and are available on request. Electronic searches were supplemented by backward and forward citation screening of all included articles and of key narrative or scoping reviews on artificial intelligence in orthodontics, as well as manual screening of recent issues of major orthodontic journals. All records were exported to a reference-management database, and duplicates were removed. Two reviewers independently screened titles and abstracts against the eligibility criteria. Any record judged potentially relevant by either reviewer progressed to full-text assessment. Full texts were then evaluated in duplicate. Disagreements at either stage were resolved through discussion, with arbitration by a third reviewer when necessary. Reasons for exclusion at the full-text stage were documented to permit transparent reporting in the flow diagram.

### Data collection

For each included study, information was recorded on publication year, country, clinical setting, study design, sample size and characteristics of the underlying patient cohort. Detailed descriptions were extracted for the AI system, including the dominant reasoning paradigm, categorized as knowledge-based systems (including rule-based, fuzzy, or Bayesian/probabilistic systems), data-driven machine-learning systems, case-based reasoning systems, or hybrid architectures, input data types (cephalometric measurements, clinical findings, radiographic features, indices of treatment need, demographic variables) and the specific decision task addressed. To improve consistency in terminology, each system was categorized according to its dominant learning or reasoning paradigm. These categories were not treated as mutually exclusive when a system incorporated more than one approach; however, each study was assigned to the paradigm that best reflected its primary architecture or mode of decision generation (Table 1).

**Table 1:** Classification of AI learning paradigms in orthodontic decision-support systems

Paradigm	Definition	Typical Methods / Models
Knowledge-based: rule-based systems	Systems based on explicitly defined clinical rules or expert-derived logic	Expert systems, deterministic decision trees
Knowledge-based: fuzzy and probabilistic systems	Systems modeling uncertainty and probabilistic relationships between clinical variables	Bayesian networks, fuzzy inference systems
Case-based	Systems that retrieve and adapt solutions from previously treated similar cases	Case-based reasoning systems
Data-driven (Machine learning)	Systems that learn patterns directly from data without explicit rule encoding	Neural networks, support vector machines, random forests, gradient boosting
Hybrid systems	Systems combining two or more paradigms within a single framework	Rule + machine learning models, case-based + machine learning systems

Where reported, comparator conditions and reference standards were recorded together with any training–test split or cross-validation strategy and whether validation was internal or external. Performance metrics, including accuracy, sensitivity, specificity, predictive values, area under the curve, confusion matrices and agreement coefficients, were extracted as presented. Any statements regarding usability, integration into clinical workflows, impact on clinicians’ decisions or educational applications were noted. Data extraction was performed independently by two reviewers and cross-checked for completeness and consistency; discrepancies were resolved by consensus.

### Risk of bias assessment

Risk of bias was appraised using tools prespecified in the protocol. In the final set of studies, all designs were non-randomized modelling or observational investigations; no randomized trials or eligible systematic reviews were identified. Consequently, the ROBINS-I tool for non-randomized studies of interventions was applied to all included studies, as the primary objective of most included studies was to evaluate AI-based systems as decision-support tools influencing clinical decision-making rather than purely to develop or validate prediction models. While tools such as PROBAST or QUADAS-2 are commonly used for prediction-model and diagnostic-accuracy studies, many included investigations combined elements of prediction, classification, and broader clinical decision support within complex orthodontic frameworks that do not align fully with these instruments. Emerging AI-specific appraisal extensions and frameworks, including PROBAST-AI, are also being developed; however, no universally accepted risk-of-bias instrument currently exists for hybrid orthodontic AI decision-support studies that integrate predictive modelling with broader clinical decision-making tasks. ROBINS-I was therefore selected as the most appropriate tool to capture bias related to participant selection, confounding, and outcome assessment in these quasi-interventional study designs.

Two reviewers independently evaluated each study across the seven ROBINS-I domains, including bias due to confounding, selection of participants, classification of interventions or exposures, deviations from intended interventions, missing data, measurement of outcomes, and selection of the reported results. Each domain was judged as low, moderate, serious or critical risk of bias, or as having insufficient information. An overall risk-of-bias judgement was then derived according to ROBINS-I guidance. Any disagreements were resolved through discussion, with recourse to a third reviewer when required. Consideration of risk-of-bias assessments informed the interpretation of findings, with greater emphasis placed on studies at low or moderate risk.

### Synthesis

Before data collection, quantitative synthesis was planned only if studies reported sufficiently homogeneous interventions, populations and outcome measures. After review of the included literature, substantial heterogeneity was identified across study design (retrospective modelling, cross-sectional, and diagnostic accuracy studies), clinical decision tasks (ranging from binary extraction decisions to multi-step treatment planning and skeletal classification), dataset characteristics (including sample size, population composition, and feature sets), and validation strategies (predominantly internal validation with limited external testing). Effect estimates were also rarely reported in a form that permitted pooling. Meta-analysis was therefore judged inappropriate and was not undertaken. Instead, a structured narrative synthesis was carried out. Studies were grouped by the dominant reasoning paradigm of the AI system, including knowledge-based systems (rule-based, fuzzy, and Bayesian/probabilistic approaches), case-based reasoning systems, data-driven machine-learning models, and hybrid frameworks. Within these groupings, ranges and patterns of performance metrics were summarized, highlighting any direct comparisons with human experts, guideline-based tools or alternative AI models. Variation in performance according to sample size, input feature set and validation strategy was described qualitatively. Evidence related to usability, clinical integration and potential educational roles was synthesized narratively. Formal assessment of small-study effects or publication bias was not attempted because of the absence of meta-analytic pooling and the small number of studies within each methodological subgroup.

## Results

**Fig. 1:**
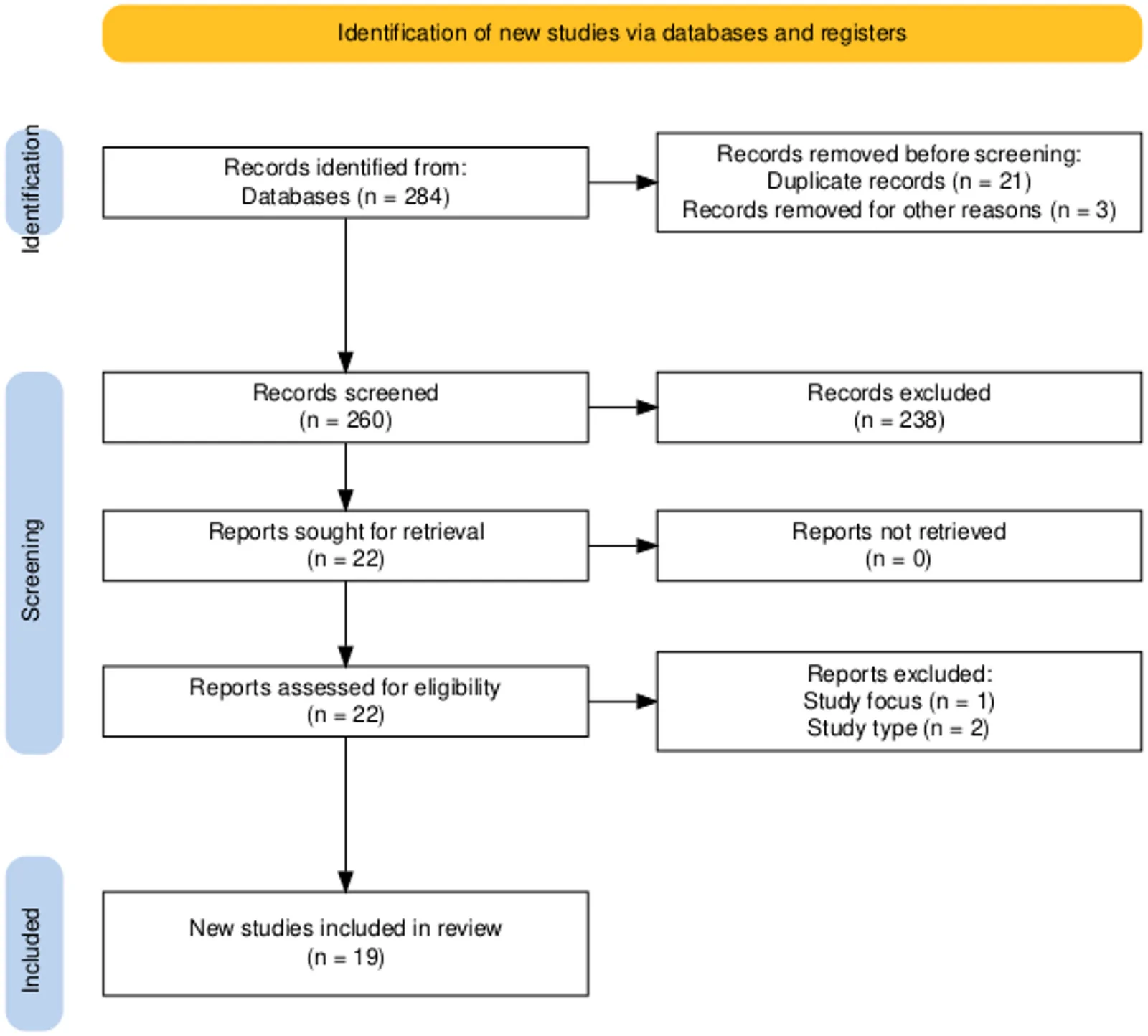
PRISMA flow diagram [34]

The database searches yielded 284 records, from which 21 duplicates and 3 records removed for other reasons were excluded before screening. The remaining 260 records were screened by title and abstract, resulting in the exclusion of 238 records that did not meet the eligibility criteria. Full texts were obtained for 22 articles, all of which were assessed for eligibility; 3 were excluded at this stage (1 due to study focus and 2 due to study type), leaving 19 studies for inclusion in the qualitative synthesis (Fig. 1). Details pertaining to the studies omitted during full text screening can be found in Supplementary Table 1.

Table 2 summarizes the clinical tasks, learning approaches, input features, and reported performance metrics of the 19 included studies. Most investigations evaluated supervised machine-learning classifiers for diagnostic or treatment-planning decisions, including artificial neural networks, support vector machines, ensemble methods, and gradient-boosted trees, while a smaller group implemented knowledge-based systems, including rule-based expert systems, fuzzy systems, Bayesian/probabilistic networks, or hybrid case-based frameworks [14, 17, 27, 28, 31, 32, 35–40]. One recent study assessed general-purpose large language models for full treatment-plan generation [41].

**Table 2:** Summary of included studies

Author(s)	Year	Clinical task / decision supported	Learning approach	Input data / predictors	Outcomes / performance
Akçam & Takada [31]	2002	Selection of appropriate extraoral headgear type (cervical / straight / high-pull) for Class I–II malocclusion	Fuzzy rule-based expert system (fuzzy inference using clinician-derived rules)	Three cephalometric variables: overjet, overbite, and SN–mandibular plane angle from lateral cephalograms	In 95.6% of cases, at least 6/8 experts judged the system’s headgear recommendation acceptable; in roughly half the cases the system exactly matched clinicians’ preferred headgear type.
Auconi et al. [26]	2015	Prediction / risk stratification of early Class III treatment outcome after rapid maxillary expansion + facemask therapy	Data-mining approach with fuzzy clustering and network analysis (unsupervised clustering into phenotypic groups)	Cephalometric variables at pre-treatment (T1) and post-growth (T2) from interceptively treated Class III patients	Three phenotypic clusters identified with markedly different failure rates: ~0% in “balanced” cluster vs. ~ 29–33% in “hypermandibular” and “hyperdivergent” clusters, allowing individualized risk estimates for treatment failure.
Jung & Kim [27]	2016	Extraction vs. non-extraction, and detailed extraction pattern, at initial orthodontic consultation	Multilayer perceptron ANN, sequential multi-step classifier (four neural networks for layered decisions)	12 cephalometric measurements + 6 clinical indices (arch length discrepancy upper/lower, molar relationship, overjet, incisor protrusion, chief complaint)	ANN achieved 93% accuracy for extraction vs. non-extraction, and 84% for detailed extraction pattern; overall sequential decision accuracy ≈ 84% (rising to ~ 97% when ambiguous “borderline” cases were excluded).
Kılıç et al. [43]	2022	Early prediction of skeletal Class III malocclusion from profile photographs	Three approaches: (1) Deep learning CNN, (2) Classical ML classifier (best = logistic regression), (3) Rule-based area-ratio method	435 profile images (Class I, II, III) with orthodontist-assigned diagnosis; image-derived features (silhouette, area ratios, facial landmarks)	Best balanced ML model (logistic regression): accuracy 76%, precision 0.74, recall 0.82, F1 0.78, AUC 0.76. Deep learning: ~70% accuracy; rule-based: ~74% accuracy.
Kim et al. [35]	2009	Prognosis prediction for Class III treatment outcome (favorable vs. unfavorable)	Support Vector Machine (SVM) with feature-wrapping for variable selection; compared to discriminant analysis and logistic regression	Pretreatment cephalometric and clinical variables; optimal subset selected by wrapper algorithm for SVM classifier	SVM with feature wrapping achieved 97.2% classification accuracy (AUC ~ 0.97), clearly outperforming discriminant analysis (84.2%) and logistic regression (86.8%) for predicting treatment success/failure.
Li et al. [36]	2019	Comprehensive orthodontic treatment planning: (1) extraction vs. non-extraction, (2) extraction pattern, (3) anchorage pattern	Three multilayer perceptron ANNs (one for each decision)	27 diagnostic variables (demographic, clinical, cephalometric) used as ANN inputs	ANN accuracy: 94.3% for extraction vs. non-extraction, 84.2% for extraction pattern, and 92.8% for anchorage pattern; authors propose this as a decision support tool to complement clinician judgement.
Mason et al. [28]	2023	Extraction vs. non-extraction decision in a racially and ethnically diverse orthodontic population	Supervised machine-learning classifiers (logistic regression, random forest, SVM, neural network), main model often reported as LR	56 predictor variables (2 demographic, 4 clinical, 50 cephalometric measurements) from pre-treatment records	Best model (logistic regression) produced AUC ≈ 0.91 with accuracy around 82% for extraction vs. non-extraction; other ML models (e.g., SVM, RF) had similar or slightly lower performance.
Midlej et al. [29]	2025	Characterisation and classification of skeletal Class I vs. II patterns in Arab orthodontic patients	Combination of unsupervised clustering (hierarchical clustering) and multiple supervised ML models (RF, CART, KNN, SVM, LDA, etc.)	28 cephalometric parameters from lateral cephalograms (skeletal and dental measurements)	General model using all parameters: random forest and CART achieved accuracy ≈ 0.87, Cohen’s κ ≈ 0.74 and AUC ≈ 0.92. Models using only ANB and Wits had lower accuracy (~ 0.78) but still “good” discrimination.
Nieri et al. [30]	2010	Factors influencing clinical management of impacted maxillary canines (surgical approach, orthodontic traction duration, periodontal outcomes)	Bayesian network (probabilistic graphical model) learned from clinical and radiographic data	Radiographic and clinical variables for impacted canines (sector location, alpha angle, vertical position, unilateral/bilateral impaction, ectopic eruption, etc.)	BN showed that bilateral impaction is strongly associated with palatal impaction and longer treatment; pre-treatment alpha-angle was the main determinant of traction duration; periodontal outcomes were largely independent of pre-treatment radiographic factors.
Prasad et al. [17]	2022	Multi-layer clinical decision support system (CDSS) for orthodontic treatment planning across several decision levels (e.g., skeletal pattern, extraction, growth modification vs. camouflage vs. surgery, anchorage & appliance choice)	Hierarchical supervised ML using several classifiers (XGBoost, random forest, decision tree, logistic regression, SVM, KNN, naïve Bayes); best model chosen per layer	33 demographic/clinical/cephalometric variables; four sequential “layers” of prediction corresponding to different diagnostic/treatment decisions	Across layers, best algorithms (often XGBoost or random forest) achieved accuracies in the mid-80s to low-90s for individual decisions; overall mean accuracy across all layers ≈ 84–85%, supporting utility as a CDSS for orthodontic planning.
Shimizu et al. [44]	2022	Automated generation of a prioritized problem list and orthodontic treatment plan from digital records	Hybrid ML system: support vector machine + self-attention (deep neural network) to model multi-label orthodontic problems and treatment steps from routine clinical records	967 orthodontic cases from one university hospital; each case had an expert-generated problem list and treatment plan. AI output compared to these labels and evaluated by independent orthodontists	For problem lists, AI achieved precision 0.65 and recall 0.55; for treatment plans, precision and recall were 0.48. In panel ranking, AI’s problem lists were rated mid-range among 4 orthodontists, while AI treatment plans performed similarly only to the lowest-rated orthodontist, indicating partial but not full clinical equivalence.
Stephens et al. [32]	1996	Decide whether cases are suitable for treatment with removable appliances in general practice vs. referral, and provide basic treatment advice	Rule-based expert system (“Jeremiah”) encoding orthodontists’ knowledge as if–then rules; no data-driven learning	20 general-practice cases (approx. half “treat”, half “refer”) assessed by 6 consultant orthodontists. Consultants rated both their own and the system’s recommendations in a Turing-test style peer review	Peer assessment showed the system’s treatment recommendations were generally judged to be of similar quality to consultant orthodontists, with referral advice considered sound though sometimes less convincing. No formal accuracy %, but overall performance lay within inter-expert variability.
Suhail et al. [37]	2020	Diagnosis of (1) extraction vs. non-extraction and (2) one of 13 detailed extraction patterns in comprehensive fixed-appliance treatment	Ensemble ML (logistic regression, random forest, gradient boosting; feature selection and stacking)	287 patients; 19 clinical and cephalometric features. Gold standard: consensus decisions of 5 experienced orthodontists on extraction need and scheme	Ensemble models showed “high performance”, with accuracies and κ statistics comparable to agreement among experts for extraction vs. non-extraction, and good but lower performance for specific extraction patterns. Key predictors included lower arch discrepancy and crowding.
Takada et al. [39]	2009	Decide to extract or not to extract (first premolars) in Class I and II cases	Mathematical / knowledge-based classifier using “feature vectors” and regression/discriminant analysis; not stochastic ML in the modern sense	188 female patients divided into extraction and non-extraction groups; 27 morphologic and cephalometric variables. Reference: decisions of a senior orthodontist	Computational model achieved an overall success rate of 90.4% in reproducing the orthodontist’s extraction vs. non-extraction decisions. Authors concluded the model could approximate expert decisions and clarify influential features.
Tanaka et al. [41]	2025	Compare AI-generated comprehensive treatment plans with expert orthodontist recommendations for a complex orthodontic case	General-purpose LLM chatbots (ChatGPT, Copilot, Bard): no additional training; free-text prompts used to generate plans	34 orthodontists each produced a treatment plan for a single case and then rated four anonymized plans (their own + 3 AI plans) on 0–100 visual analogue scales and pairwise preference	Mean VAS scores were ≈ 78 for orthodontists’ own plan vs. ≈ 47 (ChatGPT), 45 (Copilot), 38 (Bard); all AI plans scored significantly worse (*p* < 0.001). AI plans were rarely preferred and sometimes contained implausible steps, leading authors to conclude that current general-purpose LLMs are not yet reliable as autonomous orthodontic planners.
Thanathornwong [14]	2018	Clinical decision support to classify need vs. no need for orthodontic treatment in permanent dentition	Bayesian network (probabilistic graphical model) learned from patient data	1,000 permanent-dentition records from a hospital system with 15 occlusal and profile variables; BN trained on these. External test: 20 new patients examined by two orthodontists and by the BN system	Best BN model achieved specificity 100%, sensitivity 95%, accuracy 96%, AUC 0.91 on internal validation. In the 20-patient test set, agreement between the system and orthodontist A was κ = 1.00 and with orthodontist B κ = 0.894, indicating very high concordance and promising performance as a CDSS.
Wang & Yeh [40]	2010	Framework to record orthodontic patient data, retrieve similar past cases, show 3D animations, and support diagnosis/treatment planning for Class II patients	Hybrid rule-based + case-based reasoning system; knowledge-based CDSS integrated with computer-assisted instruction	Knowledge base of ~ 600 orthodontic cases; prototype focused on 66 Class II Division 1 cases with full records and imaging. Rules and cases elicited from expert orthodontists	Formal diagnostic accuracy not reported. User evaluations indicated that orthodontists found the system useful for patient education and as an adjunct for diagnosis/treatment planning; most rated the presented cases and suggested information as realistic and clinically helpful.
Xie et al. [38]	2010	Decide extraction vs. non-extraction before orthodontic treatment	Feedforward artificial neural network (23–13–1 architecture) trained with backpropagation	200 Chinese adolescents (11–15 years) with Class I malocclusion; 23 clinical/cephalometric indices used as inputs. 180 cases used for training, 20 for testing; reference decisions from an experienced orthodontist	ANN achieved 100% accuracy on the training set and 80% accuracy on the 20-case test set. Authors concluded that ANN-based models are promising tools to assist, but not replace, clinicians in extraction decision-making.
Yagi et al. [42]	2009	Decide which teeth should be extracted in cases already designated for extraction therapy	Mathematical optimization / pattern-matching model using distances from template feature vectors for different extraction patterns	193 female extraction cases with 19 morphologic traits; reference extraction patterns were those actually chosen by an expert orthodontist	Simulated extraction sites matched the orthodontist’s choice in 86.0% of cases. Analysis grouped contributing morphologic features into five patterns linked to particular extraction schemes.

A large subset of studies focused on extraction versus non-extraction decisions and detailed extraction patterns. Neural-network-based models for comprehensive extraction decision-making achieved accuracies of 93–94% for extraction versus non-extraction and around 84% for specific extraction patterns [27, 36]. Ensemble approaches that combined multiple classifiers reported accuracies and κ values comparable with agreement among expert orthodontists for the binary extraction decision, with somewhat lower performance for the 13-category extraction pattern task [37]. Additional machine-learning models developed using support vector machines, logistic regression, random forests, and related methods reported accuracies between approximately 80% and 90% and an area under the curve (AUC) of about 0.91 for extraction versus non-extraction in diverse patient samples [28, 35, 38]. Knowledge-based and mathematical optimization models designed to reproduce expert extraction decisions yielded success rates of 86–90% when compared with the original orthodontist’s chosen extraction scheme [39, 42].

Several studies addressed skeletal pattern diagnosis and risk stratification. Data-mining and clustering of cephalometric variables identified three phenotypic Class III clusters with markedly different treatment-failure rates, ranging from approximately 0% in the “balanced” group to about 29–33% in “hypermandibular” and “hyperdivergent” clusters [26]. Early prediction of skeletal Class III malocclusion from profile photographs achieved a best accuracy of 76% with logistic regression, compared with around 70% for deep learning and 74% for a handcrafted rule-based method [43]. Classification of skeletal Class I versus II patterns reached accuracies close to 0.87 with random forest and classification and regression trees (CART) models (AUC ≈ 0.92), while simplified models using only ANB and Wits values remained around 0.78 accuracy [29]. A Bayesian-network–based decision support system for treatment need classification in permanent dentition reported internal validation accuracy of 96% with 100% specificity, 95% sensitivity, and an AUC of 0.91; in a small external test set, agreement with two orthodontists ranged from κ = 0.894 to 1.00 [14]. Another Bayesian network applied to impacted maxillary canines demonstrated structural dependencies among radiographic and clinical variables, identifying pre-treatment alpha angle and bilateral impaction as key determinants of traction duration and treatment complexity [30]. A fuzzy rule-based system for headgear selection reported that, in 95.6% of cases, at least six of eight experts rated the system’s recommendation as acceptable, and in roughly half of cases the suggested headgear type exactly matched clinicians’ preferred choice [31].

Multi-layer and comprehensive decision-support frameworks were also evaluated. A four-layer machine-learning clinical decision support system using 33 demographic, clinical, and cephalometric variables achieved mean accuracies between the low 80% and low 90% range across sequential diagnostic and treatment-planning decisions, with overall average accuracy around 84–85% [17]. A hybrid rule-based and case-based environment for Class II diagnosis and planning, incorporating approximately 600 archived cases, did not report formal accuracy metrics but was rated by orthodontists as realistic and useful for both diagnosis and patient education [40]. A classic rule-based expert system for removable-appliance decisions in general practice showed that its recommendations were generally judged by consultant orthodontists to be of similar quality to peer clinicians’ plans in a Turing-test-style evaluation [32].

Finally, recent systems have attempted to automate broader components of the orthodontic treatment workflow. A hybrid support-vector-machine and self-attention model trained on 967 cases generated multi-label problem lists with a precision of 0.65 and recall of 0.55, and treatment plans with precision and recall of 0.48, with expert panels rating AI-generated problem lists in the middle of the orthodontist range and AI treatment plans comparable only to the lowest-rated orthodontist [44]. In contrast, evaluation of three general-purpose large language models for comprehensive treatment planning in a single complex case showed substantially lower visual analogue scale scores for all AI-generated plans (means 38–47) compared with orthodontists’ own plans (mean ≈ 78), and expert raters rarely preferred AI plans over human ones [41].

When considered across learning paradigms, data-driven machine-learning models generally demonstrated the highest discriminative performance, with reported accuracies frequently exceeding 80% and AUC values approaching or exceeding 0.90 in several studies [17, 28, 35, 38]. In contrast, knowledge-based systems, particularly rule-based and mathematical decision models, typically showed slightly lower but more consistent agreement with expert decisions, often in the range of approximately 85–90% when evaluated against the originating clinician’s treatment plan [32, 39, 42]. Bayesian/probabilistic and fuzzy knowledge-based systems provided moderate to high performance with the added advantage of explicit uncertainty modeling and interpretability, although their evaluation was often limited to narrower clinical tasks [14, 30, 31]. Hybrid systems showed variable performance but demonstrated potential to combine the strengths of structured reasoning and predictive modeling, particularly in multi-step decision-support frameworks [17, 40]. However, direct comparisons between paradigms were limited, and differences in study design, datasets, and validation strategies preclude definitive conclusions regarding superiority of any single approach.

Risk-of-bias assessment using ROBINS-I showed that most studies (10/19) had an overall moderate risk of bias, largely due to single-center convenience samples, retrospective designs, incomplete capture of relevant clinical factors, and limited reporting of model development and tuning (Table 3). Seven studies were judged at serious risk of bias, typically because of highly selected populations (for example, only borderline, female, or excellent-outcome cases), substantial potential for unmeasured confounding, and reliance on subjective expert judgments without adequate control or adjustment. Only one study, which used a large, clearly defined sample and robust reference standards with blinded assessment, was rated at low overall risk of bias. Measurement procedures were generally sound and based on standard orthodontic records, so measurement and misclassification bias was usually low, whereas reporting bias was commonly moderate owing to absent preregistration, incomplete description of modelling pipelines, and lack of external validation.

**Table 3:** Summary of risk of bias outcomes

Study	Selection bias	Bias due to confounding	Measurement validity / misclassification	Reporting bias	Overall RoB
Akçam & Takada, 2002 [31]	Moderate	Moderate	Moderate	Moderate	Moderate
Auconi et al., 2015 [26]	Moderate	Moderate	Low	Moderate	Moderate
Jung & Kim, 2016 [27]	Serious	Moderate	Low	Moderate	Serious
Kiliç et al., 2022 [43]	Moderate	Low-Moderate	Low	Moderate	Moderate
Kim et al., 2009 [35]	Serious	Serious	Low	Moderate	Serious
Li et al., 2019 [36]	Moderate	Moderate	Low	Moderate	Moderate
Mason et al., 2023 [28]	Moderate	Low	Low	Low-Moderate	Moderate
Midlej et al., 2025 [29]	Moderate	Low	Low	Moderate	Moderate
Nieri et al., 2010 [30]	Serious	Serious	Low	Moderate	Serious
Prasad et al., 2022 [17]	Moderate	Moderate	Low	Moderate	Moderate
Shimizu et al., 2022 [44]	Low	Low	Low	Low	Low
Stephens et al., 1996 [32]	Serious	Serious	Moderate	Moderate	Serious
Suhail et al., 2020 [37]	Moderate	Moderate	Low	Moderate	Moderate
Takada et al., 2009 [39]	Serious	Serious	Low	Moderate	Serious
Tanaka et al., 2025 [41]	Serious	Serious	Moderate	Moderate	Serious
Thanathornwong, 2018 [14]	Moderate	Low-Moderate	Moderate	Moderate	Moderate
Xie et al., 2010 [38]	Moderate	Low-Moderate	Low	Moderate	Moderate
Yagi et al., 2009 [42]	Serious	Serious	Low	Moderate	Serious

## Discussion

This review set out to map how artificial intelligence and knowledge-based systems have been applied as clinical decision support for orthodontic diagnosis and treatment planning, to assess how closely their recommendations approximate those of expert clinicians, and to explore how different reasoning approaches influence performance and interpretability. Nineteen primary studies were identified, largely retrospective model-development or validation investigations conducted in academic environments. AI systems were most commonly deployed to support extraction versus non-extraction decisions or the selection of extraction patterns, to predict the prognosis of growth-modification or camouflage, to classify skeletal relationships, or to generate broader, multi-layered treatment plans [17, 26–28, 31, 35–38]. Across this heterogeneous evidence base, most systems reported high apparent accuracies, often exceeding 80%, for reproducing expert decisions related to extraction need, skeletal classification, and broad treatment-modality selection. However, these performance estimates should be interpreted cautiously. Most studies were at moderate to serious risk of bias, only a minority used rigorous internal validation, and very few were prospectively evaluated in clinical settings. Retrospective designs, highly selected samples, limited external validation, and reliance on expert opinion as the reference standard may therefore have inflated reported model performance [17, 28, 41, 44].

### Interpretation by learning and reasoning approach

In relation to the first research question, how different AI decision-support approaches have been used to support orthodontic treatment planning, clear differences emerge between learning paradigms in terms of performance, interpretability, and clinical applicability. Broadly, knowledge-based systems, including rule-based, fuzzy, and Bayesian/probabilistic approaches, prioritize interpretability and alignment with clinical reasoning. In contrast, data-driven machine-learning models emphasize predictive performance, often at the expense of transparency. Hybrid systems attempt to bridge these trade-offs, although their evaluation remains limited. Early rule-based and expert-system work demonstrated that orthodontic diagnostic logic can be encoded explicitly as if–then rules, with examples including the “Jeremiah” system for fixed-appliance planning and a framework that combined rule logic with case retrieval and educational animations for Class II problems [32, 40]. These systems emphasized transparency and explainability. However, they required intensive knowledge engineering and were often difficult to extend to new clinical questions or practice styles.

Fuzzy and Bayesian/probabilistic knowledge-based systems attempted to better reflect uncertainty and gradations in clinical judgement. Fuzzy rules have been used to recommend headgear type from cephalometric inputs so that borderline cases can be handled in a graded rather than binary way [31]. Bayesian networks have been used for treatment-need classification with probabilistic outputs that support explicit thresholding for care. They have also been applied to impacted maxillary canines, where they characterize relationships among predictors such as sector position, angulation, and vertical distance from the occlusal plane [14, 30]. Collectively, these knowledge-based approaches favor interpretability and alignment with established theory but tend to be narrow in scope and dependent on the assumptions of the experts who construct them.

Machine-learning models for extraction decisions form the largest subgroup of studies and speak directly to the second research question regarding performance relative to expert decisions. Early work used support vector machines or feed-forward neural networks to predict prognosis for Class III treatment or the need for extraction, reporting very high in-sample accuracies, including near-perfect discrimination in some analyses [35, 38]. Subsequent studies formalized extraction decisions in two stages: first whether extraction was required, and then which teeth should be removed. These approaches combined mathematical pattern-matching and discriminant analysis, helping bridge knowledge-based reasoning and data-driven classification while clarifying which morphologic features influenced extraction patterns [39, 42].

More recent work has adopted multilayer perceptrons and ensemble methods for extraction versus non-extraction and detailed extraction schemes, achieving accuracies in the low- to mid-80% range for global extraction decisions and slightly lower for specific patterns, broadly comparable to agreement levels among experienced orthodontists [27, 28, 37]. In a racially and ethnically diverse cohort with an extensive set of cephalometric predictors, one such model achieved an AUC of approximately 0.91, suggesting that well-designed machine-learning systems can perform robustly across heterogeneous populations when training data are sufficiently representative [28]. Nonetheless, almost all extraction models are trained solely to reproduce expert decisions; none links predictions to objective long-term treatment outcomes, and calibration, decision-curve analyses and effects on patient-centered outcomes have not been reported.

Several studies extended beyond single decisions to skeletal classification and prognosis. Fuzzy clustering and network analysis have been used to define phenotypic clusters with markedly different failure risks in interceptively treated Class III patients, while a range of machine-learning algorithms have been applied to classify skeletal Class III or distinguish Class I from Class II patterns using cephalograms and profile images [26, 29, 43]. These investigations indicate that pattern-recognition methods can augment clinicians’ ability to classify skeletal relationships early and potentially guide decisions about growth modification versus camouflage or surgery, although direct evidence for improved clinical outcomes remains limited.

Comprehensive decision-support systems address the third research question concerning the influence of knowledge representation on usability and clinical integration. Neural-network and layered machine-learning architectures have been used to predict multiple components of the treatment plan—extraction decisions, extraction patterns, anchorage strategies and treatment modalities for different skeletal bases—within a single framework [17, 36]. These systems move closer to realistic clinical workflows and illustrate how AI may propose coherent multi-step treatment strategies rather than isolated decisions. However, they remain trained largely on previous clinician choices and may therefore reproduce existing practice patterns rather than objectively validated or outcome-based care.

Two studies explicitly compared AI outputs with clinician performance in realistic evaluation settings. Validation of commercial AI tools showed that AI-generated problem lists were rated similarly to those of mid-range orthodontists, whereas treatment plans lagged behind the best clinicians [44]. In an experimental comparison of comprehensive plans generated by large language models against orthodontists’ own plans, the AI outputs were scored substantially lower and sometimes contained clinically implausible steps [41]. Together, these findings suggest that domain-specific AI trained on structured orthodontic data can achieve partially acceptable performance, whereas current general-purpose language models without orthodontic fine-tuning are not yet suitable as autonomous planners and should be used, if at all, only as adjuncts.

### Methodological considerations and risk of bias

Risk-of-bias assessment indicated substantial methodological limitations in the underlying evidence. Most studies were judged at moderate overall risk of bias, with several at serious risk, primarily because of participant selection and confounding. Samples were typically drawn from single university clinics. Some analyses further restricted the population to patients with excellent outcomes or specific demographic characteristics, raising concerns about poor generalizability to other settings, treatment philosophies, or patient groups. Experimental diversity was limited; syndromic cases, severe asymmetries and patients with significant comorbidities were commonly excluded, yet these are precisely the situations in which additional decision support might be most valuable.

Although confounding is less central for prediction modelling than for causal inference, several models relied on narrow sets of morphologic variables and omitted contextual information such as patient preferences, periodontal condition, caries risk or socioeconomic factors, which may constrain model usefulness and introduce systematic bias. Overfitting is also a concern: some of the highest reported accuracies came from very small datasets combined with complex algorithms and minimal test sets, making it likely that performance would fall on truly independent data. Internal validation was often limited to simple split-sample evaluation, and only a few studies reported cross-validation or any form of external testing.

Outcome measurement frequently used orthodontists’ treatment decisions or prioritized problem lists as the reference standard. This represents a fundamental limitation of the current evidence base, as most AI systems are trained and evaluated to reproduce expert opinion rather than objective clinical outcomes or long-term treatment success. Although pragmatic, this approach means that AI models primarily learn to reproduce local expert preference rather than objective treatment quality, universally accepted treatment standards, or long-term clinical outcomes.Inter-rater reliability among experts was seldom quantified, and few studies explored how disagreement between clinicians influences model training or evaluation. Reporting bias and incomplete methodological transparency were common. Many articles described only the best-performing algorithms and provided limited detail regarding feature selection, hyper-parameter tuning, handling of missing data, or class imbalance. None adhered fully to emerging reporting standards for AI prediction models, which complicates critical appraisal and replication.

Given these limitations, the overall certainty of evidence for the effectiveness of AI-based clinical decision support in orthodontic treatment planning should be regarded as low. The high apparent accuracy reported across studies is likely influenced by study design limitations and may overestimate real-world performance. In addition, although tools such as PROBAST, emerging AI-specific extensions (e.g., PROBAST-AI), and QUADAS-2 are commonly recommended for prediction and diagnostic-accuracy studies, the included investigations frequently combined predictive modelling with broader clinical decision-support objectives. The use of ROBINS-I therefore allowed for assessment of bias domains relevant to clinical decision-making, although it may not fully capture all sources of bias specific to machine-learning model development.

### Implications for practice and research

Taken together, current evidence suggests that AI-based decision-support systems, including knowledge-based and data-driven approaches, can approximate expert orthodontic decisions for well-defined tasks such as extraction need, skeletal classification and broad treatment-modality selection, particularly when trained on sufficiently rich local data [17, 27, 28, 36–38]. Knowledge-based systems, including rule-based systems and Bayesian/probabilistic, provide interpretable decision logic and may be useful for teaching, standardizing departmental protocols and offering “second opinions” for complex cases [14, 30–32, 40]. Data-driven machine-learning and ensemble models, including those implemented as multi-layer clinical decision-support systems, offer greater flexibility and often higher discriminative performance but at the cost of increased opacity and susceptibility to dataset bias [17, 26, 29, 43]. At present, these systems are best viewed as adjunctive tools that can support, but not replace, clinician judgement, particularly in atypical or high-stakes cases. Implementation of AI decision support in orthodontics will require careful attention to integration with electronic health records and imaging systems, workflow design and clinician training, as illustrated by early frameworks that explicitly address usability and educational value [32, 40]. Ethical considerations include the need for explainability in life-altering decisions such as orthognathic surgery versus camouflage. Additional concerns include algorithmic bias across demographic groups, the importance of diverse training cohorts, and robust governance of patient data used for AI development [28].

Future research should prioritize prospective, multicenter external validation of promising models, including those for comprehensive treatment planning and early skeletal risk stratification. Randomized or quasi-experimental studies that assess the impact of AI-assisted planning on clinician decisions, treatment efficiency, objective treatment outcomes and patient-reported measures are needed, building on early work comparing AI-generated and clinician plans. Methodological reporting should follow dedicated prediction-model guidelines, with clear description of datasets, feature engineering, model architectures, calibration and clinical-utility analyses. Hybrid systems that combine interpretable rule-based or Bayesian components with flexible machine-learning algorithms may offer a promising path to balance performance with transparency, as suggested by preliminary work integrating fuzzy rules, Bayesian networks and ensemble learners.

In practical clinical settings, current AI-based decision-support systems may be most useful in well-defined, high-variability scenarios. These include supporting extraction versus non-extraction decisions in borderline cases, assisting early skeletal classification and risk stratification, and providing structured second opinions during treatment planning. Knowledge-based and case-based systems may also have particular value in educational contexts, where their transparency can support training and standardization of clinical reasoning. In contrast, fully autonomous or comprehensive treatment planning remains beyond the capabilities of current systems.

## Conclusions

Artificial intelligence decision-support systems in orthodontics, including knowledge-based, data-driven, and hybrid approaches, can reproduce many elements of expert decision-making, particularly for extraction decisions and broad treatment-modality selection. Across the available literature, these tools generally demonstrate high apparent diagnostic and planning accuracy when tested against specialist treatment plans. However, most included studies were at moderate to serious risk of bias. These performance estimates should therefore be interpreted cautiously. Collectively, these findings suggest meaningful potential for AI-assisted clinical decision support in orthodontics. Furthermore, most evidence derives from retrospective modelling studies at moderate to serious risk of bias, with only very limited randomized or prospective evaluation. At present, AI should therefore be regarded as an adjunctive aid that can provide structured second opinions and educational support rather than an autonomous decision maker. Robust, prospectively designed studies and trials that evaluate patient-centered outcomes, model calibration and real-world impact are required before routine integration of such systems into everyday orthodontic practice can be recommended. 

## Supplementary Information

Supplementary Material 1: Table S1. Studies excluded at full-text screening stage.

## Data Availability

All data analyzed during this systematic review are included in this published article and its supplementary information files. No new datasets were generated during the current study.

## Abbreviations

AI: Artificial intelligence
AUC: Area under the curve
CART: Classification and regression trees
CDSS: Clinical decision support systems
ML: Machine learning
