# Supplementary material for: AI in orthodontic treatment planning: a systematic review comparing learning approaches
Source: BMC Oral Health. 2026 Jun 12;26:1677. doi: 10.1186/s12903-026-08854-x (PMC13551705; doi:10.1186/s12903-026-08854-x)
Supplement: Supplementary file 1 [file 12903_2026_8854_MOESM1_ESM.docx]

**Supplementary Table 1.** *Studies excluded at full-text screening stage*

| **Author(s)** | **Year** | **Title** | **Exclusion Reason** |
| --- | --- | --- | --- |
| Bahaa et al. | 2011 | The Artificial Intelligence Approach for Diagnosis, Treatment and Modelling in Orthodontic | Study type |
| Koole et al. | 2015 | Using clinical cases to stimulate active learning in a short periodontal continuing professional development course | Study focus |
| Surendran et al. | 2024 | The Future of Orthodontics: Deep Learning Technologies | Study type |
